# ^1^H, ^15^N, ^13^C resonance assignment of human osteopontin

**DOI:** 10.1007/s12104-014-9594-7

**Published:** 2015-01-24

**Authors:** Gerald Platzer, Szymon Żerko, Saurabh Saxena, Wiktor Koźmiński, Robert Konrat

**Affiliations:** 1 Max F. Perutz Laboratories, Department of Computational and Structural Biology, University of Vienna, Campus Vienna Biocenter 5, 1030 Vienna, Austria; 2 Faculty of Chemistry, Biological and Chemical Research Centre, University of Warsaw, Żwirki i Wigury 101, 02-089 Warsaw, Poland

**Keywords:** Osteopontin, Intrinsically disordered protein, Extracellular matrix, Biomineralization

## Abstract

Osteopontin (OPN) is a 33.7 kDa intrinsically disordered protein and a member of the SIBLING family of proteins. OPN is bearing a signal peptide for secretion into the extracellular space, where it exerts its main physiological function, the control of calcium biomineralization. It is often involved in tumorigenic processes influencing proliferation, migration and survival, as well as the adhesive properties of cancer cells via CD44 and integrin signaling pathways. Here we report the nearly complete NMR chemical shift assignment of recombinant human osteopontin.

## Biological context

Osteopontin (OPN) is an intrinsically disordered protein (IDP) with a molecular mass of the recombinant version around 33.7 kDa. It is highly negatively charged (25 % of the protein are glutamic or aspartic acid residues) and target of several post translational modifications including phosphorylation and glycosylation (Christensen et al. 2005). OPN is involved in a multitude of physiological and pathophysiological processes and a wide variety of cell types express OPN (Kunii et al. 2009). In bone, both matrix-synthesizing osteoblasts and bone-resorbing osteoclasts secrete OPN into the extracellular space where the protein controls calcium mineralization and the attachment of osteogenic cells to the bone matrix. OPN can also add physical strength to the extracellular matrix (ECM) since it is being cross-linked by transglutaminase to other ECM-proteins, for example collagen (Kaartinen et al. 1999). OPN is also expressed by activated immune cells such as macrophages, T-cells and B-cells, acting as a cytokine and homing these cells to sites of inflammation or injury (Ashkar et al. 2000). Epithelial cells (breast, urinary tract, gall bladder..) secrete OPN into biological fluids like blood, urine and milk. OPN is also localized to the luminal surfaces of these cells suggesting a protective role against unwanted interactions with the environment (Brown et al. 1992). Aside it’s physiological role, OPN is overexpressed in a variety of malignant tissues exerting its tumorigenic function by interacting with CD44 receptors and several integrin variants through RGD-dependent (Arginine-Glycine-Aspartic Acid) and independent mechanisms altering cell signaling events that ultimately lead to the formation of metastases (Rangaswami et al. 2006).

## Methods and results

### Protein expression and purification

The coding region of human Osteopontin (hOPN), excluding the first 16 N-terminal amino acids composing the signal peptide sequence, was amplified by PCR from a mammalian expression vector pDNR-LIB (Thermo Scientific Bio-Clone ID: 3828885) introducing a 5′ NotI and a 3′ NcoI restriction site. The obtained fragment was inserted in-frame into the bacterial expression vector pet-M11 (Pinotsis et al. 2006) yielding pet-M11-hopn coding for human hOPN fused to an N-terminal His6-Tag with a TEV-cleveage site separating the coding region of hOPN from the tag. ^15^N/^13^C labeled protein was expressed in the *E. Coli* strain BL21(DE3) (New England Biolabs) in isotopically labeled minimal media following the protocol of Marley et al. (2001). Protein expression was induced at an A_600 nm_ of 0.8 by addition of a final concentration of 1 mM IPTG. Cells were harvested after 16 h of expression at 28 °C by centrifugation at 4,500 rpm for 20 min. The bacterial pellet was resuspended 20 ml of 1xPBS low Imidazole buffer (140 mM NaCl, 2.7 mM KCl, 10 mM Na_2_HPO_4_, 1.8 mM KH_2_PO_4_ and 20 mM Imidazole) per liter of bacterial culture. Bacterial cells were lysed by sonication and subsequently subjected to boiling (10 min at 95 °C) removing all heat-unstable impurities including proteases. The obtained cell lysate was cleared by centrifugation at 18,000 rpm for 20 min. The supernatant containing the soluble protein fraction was loaded onto a 6 ml HisTrap FF Crude (GE Healthcare) affinity column pre-loaded wit Ni^2+^. After loading, the column was washed with 10 volumes of 1xPBS low Imidazole buffer before elution with 1xPBS high Imidazole buffer (140 mM NaCl, 2.7 mM KCl, 10 mM Na_2_HPO_4_, 1.8 mM KH_2_PO_4_ and 500 mM Imidazole) using a step gradient. The collected fraction containing H6-hOPN was then loaded onto a ResourceQ 6 ml ion exchange column (GE Healthcare). After a wash step with 10 column volumes of 1xPBS buffer (140 mM NaCl, 2.7 mM KCl, 10 mM Na_2_HPO_4_, 1.8 mM KH_2_PO_4_), the protein was eluted using 1xPBS high salt buffer (1 M NaCl, 2.7 mM KCl, 10 mM Na_2_HPO_4_ and 1.8 mM KH_2_PO_4_) with a linear gradient over 10 column volumes. Fractions containing the protein were pooled and concentrated to 500 μl using a 15 ml Amicon Centrifugal Filter Unit 10000MWCO (Millipore). TEV cleavage was performed in 1xPBS cleavage buffer (140 mM NaCl, 2.7 mM KCl, 10 mM Na_2_HPO_4_, 1.8 mM KH_2_PO_4_, 1 mM DTT and 1 mM EDTA) by incubating H6-hOPN with 1 mg of TEV protease for every 50 mg of protein for 16 h at 4 °C. A final ion-exchange step was used to remove the cleaved HisTag and TEV protease. hOPN was concentrated to a final concentration of 0,55 mM in phosphate buffer (50 mM NaCl and 50 mM sodium phosphate, pH 6.5) for subsequent NMR analysis.

### NMR experiments

All spectra were acquired at 293 K on an Agilent Direct Drive 700 MHz spectrometer using the standard 5 mm ^1^H–^13^C–^15^N triple-resonance probe head. The backbone ^1^H, ^13^C and ^15^N resonances were assigned using sparse random sampling of indirectly detected time domains, in order to increase resolution. A 3D HNCO experiment was used as a base spectrum for SMFT (Sparse Multidimensional Fourier Transform) processing of higher dimensionality experiments (Kazimierczuk et al. 2009). Backbone assignment was achieved using 5D HN(CA)CONH (Kazimierczuk et al. 2010), (HACA)CON(CA)CONH (Zawadzka-Kazimierczuk et al. 2012b), (H)NCO(NCA)CONH (Zawadzka-Kazimierczuk et al. 2012b) and HNCOCACB (Zawadzka-Kazimierczuk et al. 2012b) experiments. Side-chain assignments were obtained using 5D HabCabCONH (Kazimierczuk et al. 2010) and HC(CC-tocsy)CONH (Kazimierczuk et al. 2009; Hiller et al. 2008) experiments.

All NMR data sets were processed by multidimensional Fourier transformation using the home written software package (http://nmr.cent3.uw.edu.pl/software). The resonance assignment was performed using the TSAR program (Zawadzka-Kazimierczuk et al. 2012a). The input data for TSAR was prepared using Sparky (Goddard and Kneller 2002).

### Extent of assignment and data deposition

**Fig. 1:**
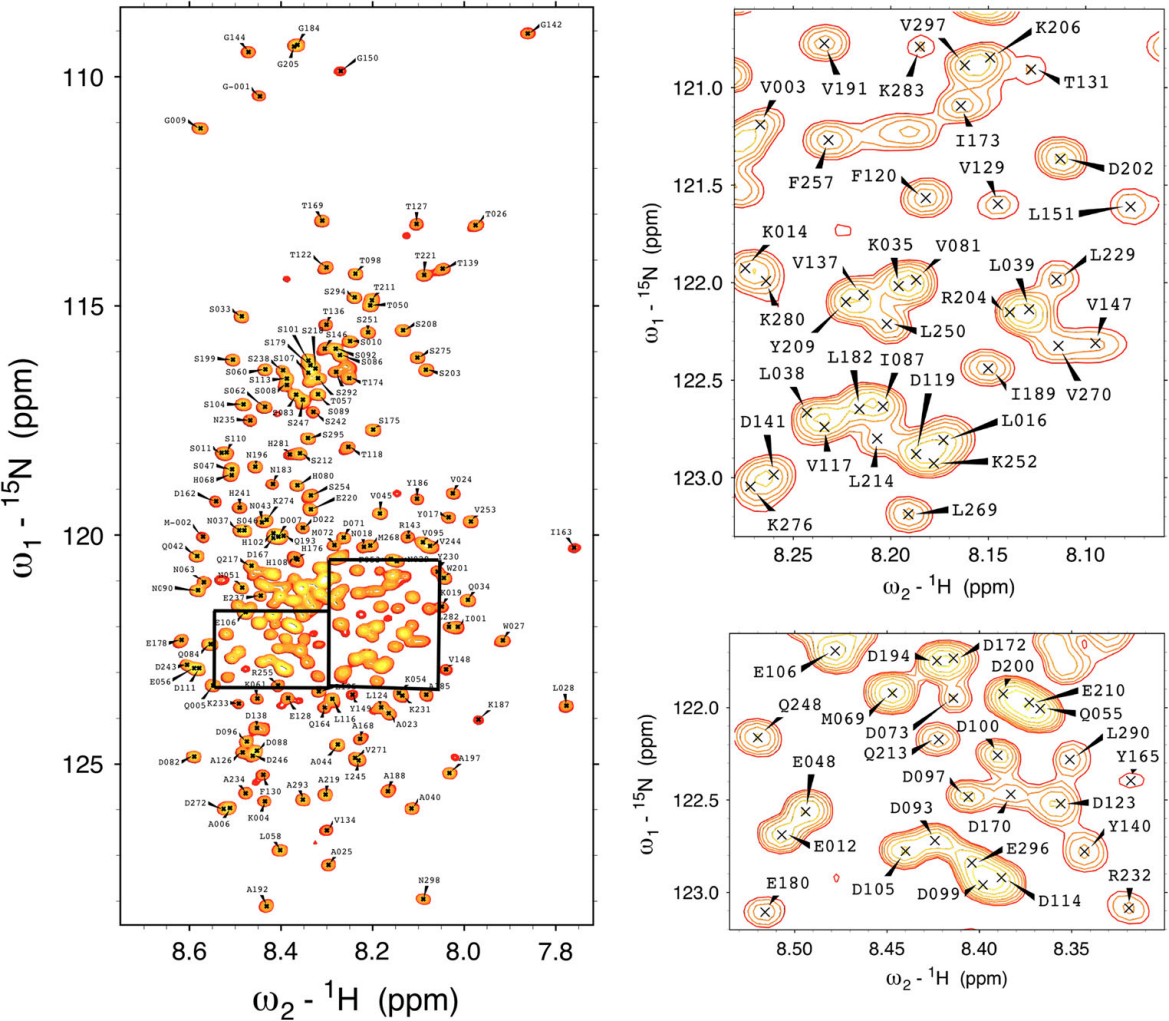
^1^H–^15^N HSQC spectrum of hOPN at pH6.5 at 293 K. For clarity reasons, the labeling in the highly crowded aspartic and glutamic acid rich region has been omitted in this figure

**Fig. 2:**
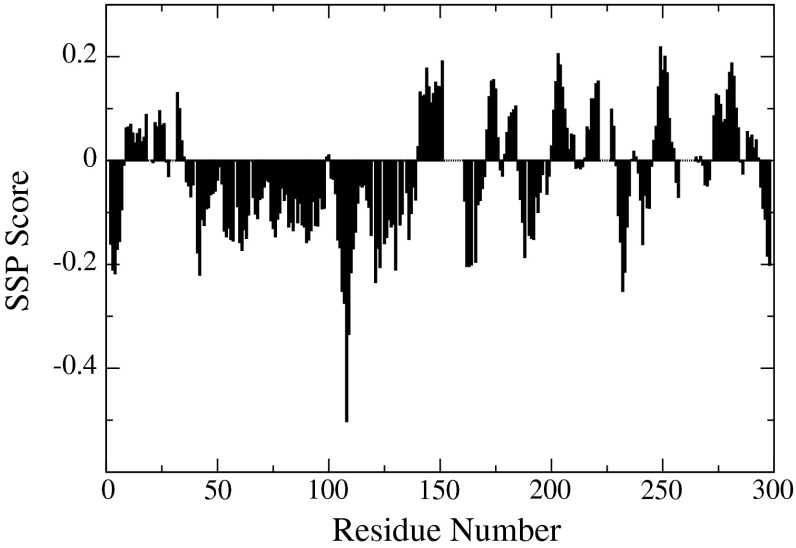
Secondary structure propensity (SSP) scores for hOPN using NH, HN, Cα, Cβ and CO chemical shifts. Positive values represent α-helical propensities and negative values represent extended or β-strand propensities

As expected for an intrinsically disordered protein the proton chemical shift dispersion of the ^1^H–^15^N HSQC spectrum (Fig. 1) shows a narrow profile with chemical shift values close to random coil values. Despite being classified as an IDP, a thorough characterization of a quail OPN homologue shows that the protein partially occupies stably folded substructures (Platzer et al. 2011; Kurzbach et al. 2013). Extensive signal overlap in conventional 2D & 3D spectra could be overcome by using the aforementioned 5D experiments. Additionally, signal assignment was supported by 3D HNN and HN(C)N experiments (Panchal et al. 2001). Notably, several segments of the protein remained undetectable. Presumably, the high percentage of basic residues in the missing regions (152–160, 222–226, 258–265) gives rise to an increased hydrogen exchange rate for the backbone amides leading to exchange broadening below the limit of detection (Molday et al. 1972). In total 90 % of backbone ^15^N, 90 % of ^1^HN, 88 % of ^13^Cα, 87 % of ^13^Cβ and 83 % of ^13^C′ resonances have been assigned. Additionally, HC(CC-tocsy)CONH spectra allowed the unambiguous assignment of 46 % side-chain carbons and 12 % side-chain protons. The SSP (secondary structure propensity) score (Marsh et al. 2006) only shows small deviations from random coil values (Fig. 2) with a long conformationally extended stretch (negative index values) in the N-terminus of the protein.

The ^1^H, ^13^C and ^15^N chemical shifts have been deposited in the BioMagResBank (http://www.bmrb.wisc.edu/) under the BMRB accession number 19999.
